# Exploring oncology treatment strategies with tyrosine kinase inhibitors through advanced 3D models (Review)

**DOI:** 10.3892/mi.2024.212

**Published:** 2024-12-20

**Authors:** Giorgia Isinelli, Sharon Failla, Roberto Plebani, Alessandro Prete

**Affiliations:** 1 Department of Cancer Biology, Dana Farber Cancer Institute, Boston, MA 02115, USA; 2 Department of Chemistry, Biology and Biotechnology, University of Perugia, I-06123 Perugia, Italy; 3 Department of Biomedical and Biotechnological Sciences, University of Catania, I-95123 Catania, Italy; 4 Department of Medical, Oral and Biotechnological Sciences, ‘G. D'Annunzio’ University, I-66100 Chieti-Pescara, Italy; 5 Department of Clinical and Experimental Medicine, Endocrine Unit 2, University of Pisa, I-56122 Pisa, Italy

**Keywords:** tyrosine kinase inhibitors, 3D models, oncology, tumor microenvironment

## Abstract

The limitations of two-dimensional (2D) models in cancer research have hindered progress in fully understanding the complexities of drug resistance and therapeutic failures. However, three-dimensional (3D) models provide a more accurate representation of *in vivo* environments, capturing critical cellular interactions and dynamics that are essential in evaluating the efficacy and toxicity of tyrosine kinase inhibitors (TKIs). These advanced models enable researchers to explore drug resistance mechanisms with greater precision, optimizing treatment strategies and improving the predictive accuracy of clinical outcomes. By leveraging 3D models, it will be possible to deepen the current understanding of TKIs and drive forward innovations in cancer treatment. The present review discusses the limitations of 2D models and the transformative impact of 3D models on oncology research, highlighting their roles in addressing the challenges of 2D systems and advancing TKI studies.

## 1. Introduction

Two-dimensional (2D) cell cultures have long been used in oncology to study cancer behaviors and drug responses (1). However, these models fall short in replicating the complex interactions of the tumor microenvironment (TME), limiting their ability to accurately predict therapeutic outcomes and resistance mechanisms. As a result, the translational potential of preclinical findings based on 2D systems is constrained. To address these limitations, three-dimensional (3D) models have emerged, providing a more realistic simulation of *in vivo* tumor dynamics. By capturing key aspects of cellular architecture and TME interactions, 3D models enhance our understanding of cancer biology and improve the evaluation of chemotherapeutic agents, particularly tyrosine kinase inhibitors (TKIs). The present review discusses the limitations of 2D models and the transformative impact of 3D models on oncology research, highlighting their roles in overcoming the shortcomings of 2D systems and advancing TKI studies.

## 2. Limitations of 2D models in assessing chemotherapeutic drug responses

As presented in Table I, 2D models present multiple limitations. The evaluation of pharmacokinetics is limited in 2D models, as they often neglect crucial aspects, such as drug metabolism, distribution and excretion. For instance, penetration into tumor tissues and interactions with surrounding cells and extracellular matrix (ECM) components remain inadequately understood with 2D models (2). These elements, indeed, play a vital role in determining drug efficacy and toxicity but pose challenges for accurate assessment within 2D culture systems.

The failure to replicate drug resistance mechanisms is another significant drawback of 2D models. Tumor cells can develop resistance to chemotherapy via various pathways, including genetic mutations, epigenetic alterations and interactions with the surrounding microenvironment (3). Moreover, 2D models typically lack key physiological aspects, such as gradients of oxygen, nutrients and signaling molecules inherent in complex biological systems. These gradients play fundamental roles in influencing cellular responses to drugs, including developing resistance mechanisms (4). All these complexities may not be faithfully represented in 2D models and lead to erroneous predictions of drug effectiveness (5,6). The primary constraint of 2D models lies in their diminished predictive precision concerning drug reactions and adverse effects in humans.

The 2D models lack the 3D arrangement and cellular interplay found *in vivo*, such as ECM, immune cells and blood vessels, which can significantly affect the study of drug interactions in a physiologically relevant context (7-10). Although they provide valuable insight into fundamental cellular mechanisms, their capacity to faithfully recreate intricate *in vivo* circumstances remains constrained. Emerging evidence from recent studies suggests that augmenting the dimensionality of the ECM surrounding cells and transitioning from 2D to 3D models can profoundly influence cell proliferation, differentiation, cell survival, and, most importantly, cellular responses to external stimuli and challenges (11-15). The absence of mechanical forces, such as shear stress and compression, which are prevalent in the TME, further limits the relevance of 2D models in recapitulating *in vivo* conditions (16,17). With regards to this, various studies have used models able to respond to mechanical stimuli (18-20). In the study by Gill and West (21), it was demonstrated that their model was able to respond to mechanical stimuli and alter the bioactivity. Several models have been proposed to optimize the ECM composition and cell interactions, modeling tissues with higher fidelity and providing more suitable platforms to be used in drug testing and cancer treatment response (22-24) as shown by Mukubou *et al* (23) in a xenograft model with gemcitabine and radiotherapy treatment. For example, in anchorage-dependent cells, adhesive interactions with the extracellular matrix and neighboring cells are vital for determining the shape, spatial organization, gene expression, proliferation rate, response to stimuli and drug metabolism. These factors collectively regulate the close association between cell structure, signaling and function (25,26).

Numerous aspects of tumorigenesis and metastasis are frequently oversimplified in monocultures; for example, Riedl *et al* (27) proved that 3D models exhibited more potent antitumor responses to AKT/mTOR/S6K or MAPK pathway inhibitors compared to 2D models. In 2D cultures, blocking AKT/mTOR/S6K increased ERK phosphorylation; however, in 3D spheroids, ERK signaling was reduced under the same conditions (27). Consequently, 2D cultures often do not adequately recapitulate the complex TME, diffusion gradients, and cellular attributes characteristic of *in vivo* systems. This disparity contributes to deviations from the anticipated response observed in animal and computational modeling, as well as during clinical testing (13,28).

False positives or negatives may occur in drug screening when compounds, that appear effective in simplistic 2D models, fail to translate to success in more complex *in vivo* models or clinical trials. Horvath *et al* (29) noted that the recurrent inability to translate promising preclinical drug candidates into clinical success underscores the limited efficacy of current disease models employed in drug discovery. A clear reluctance to explore and embrace alternative cell- and tissue-based model systems, along with a disconnect from clinical practice during assay validation, contributes to ineffective translational research. As personalized medicine seeks to customize treatments according to individual patients' distinct genetic and molecular characteristics, 2D models may fall short in capturing the specific factors influencing drug responsiveness (30). This limitation curtails their effectiveness in personalized medicine strategies. For example, in ovarian cancer, the introduction of 3D models has allowed for the identification of various important features, as demonstrated by Kerslake *et al* (31). The top enriched gene sets for 2D vs. 3D included IFN-α and the IFN-γ response, TNF-α signaling, IL-6-JAK/STAT3 signaling, angiogenesis, hedgehog signaling, apoptosis, epithelial-mesenchymal transition (EMT), hypoxia and the inflammatory response (31).

Numerous investigations have underscored that the intricacies of tissue organization, differentiation and gene expression are more faithfully portrayed in 3D cell cultures (32,33). The configuration displayed in 3D models facilitates the growth of cells within an environment that maintains spatial complexities akin to *in vivo* conditions, enabling cells to differentiate and interact in a tissue-specific manner (34). In the field of cancer, interactions between malignant cells and stromal components within the TME, comprising both cancerous and non-cancerous cellular and non-cellular elements, are crucial in influencing tumor initiation, progression, and response to treatment. This reciprocal communication profoundly influences tumor behavior (35).

The present review discusses how 3D models have aided in the understanding of actual TKI treatment and their resistance mechanisms, owing to the ability to study the TME in a more detailed manner.

## 3. Most frequently used 3D models

**Figure 1:**
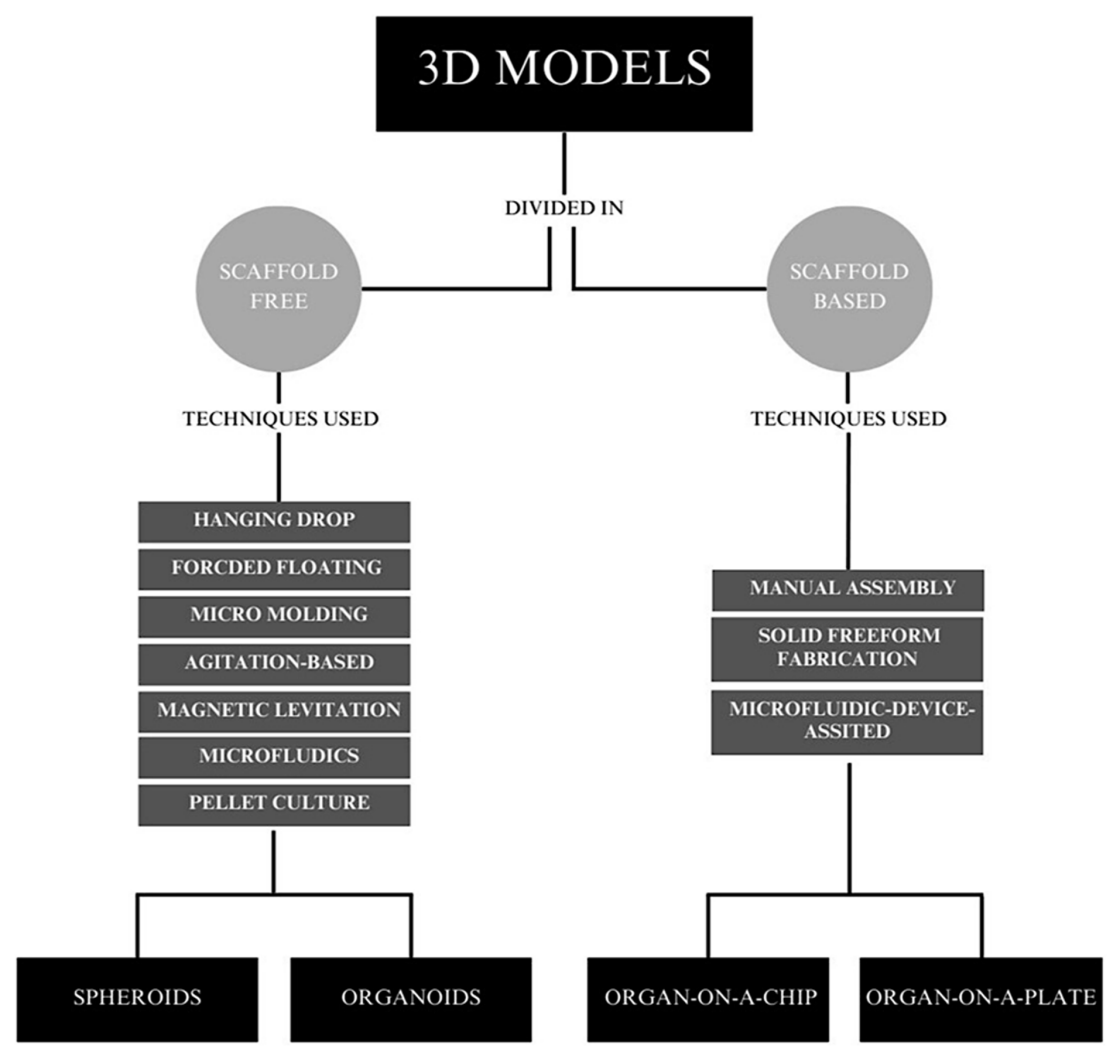
Categorization of various 3D models based on the presence of scaffolds and the techniques used for their production. The figure is divided into two primary sections: scaffold-based 3D models and scaffold-free 3D models. Each section further categorizes the models according to the specific techniques utilized in their fabrication.

The analysis of the TME plays a pivotal role in investigating novel anticancer therapies, particularly in elucidating mechanisms of resistance to immunotherapies. Utilizing 3D cell culture models enables the comprehensive analysis of tumor cell architecture and composition, facilitating the identification of potential responders to immunotherapy. 3D models, on the other hand, reproducing the TME more accurately, represent an exciting alternative in experimentation (36). Through 3D models, it is possible to perform more detailed experiments, often bridging the gap between 2D cell cultures and animal models (37). Ideally, a 3D model should simulate the specific microenvironment of a pathophysiological condition. However, the currently available models do not entirely fulfill these criteria, each presenting its strengths and limitations. Therefore, it is advisable to select the 3D model most appropriate to the specific need. 3D models can be divided into those with or without scaffolding (Fig. 1). In the first case, there is no need for an external support structure. In the second case, the seeding and cultivation of cells take place via structures that provide physical support. Models equipped with a scaffold can more easily imitate the interactions between the cell and the ECM, while models without a scaffold, such as spheroids, depending on their size, are more susceptible to physiological as well as cellular gradients (18,35).

### Scaffold-free 3D models: Spheroids and organoids

Currently, several techniques do not use the support of a scaffold to generate a tissue of interest. The first method developed was the suspended drop technique introduced in 1944 for the culture of embryonic stem cells. This technique is now mainly used for the generation of spheroids. Scaffold-free culture methods are therefore based on the ability to form a specific tissue, exploiting cellular self-aggregation, thus producing a 3D matrix (38). The use of scaffold-free methods, through a high yield, provides the possibility of combining different cell types while exploiting simple methodologies (39). Among the techniques used for the production of scaffold-free 3D models, it is possible to include hanging drop, forced floating, micro molding, agitation-based techniques, magnetic levitation, microfluidics and pellet culture (40) (Fig. 1): i) In the hanging drop method, a single spheroid is formed following the accumulation of cells at the liquid-air interface due to the inversion of the tray in the plate. ii) The forced floating method results in high-performance data, using plates coated with hydrophilic material or completely uncoated (41); iii) in the micro molding techniques, micro molds used are generally made of agarose, in which the cells are seeded and left to self-aggregate (42). iv) Agitation-based techniques use bioreactors; the latter is equipped with a chamber that is filled with a cell suspension of appropriate density, which is constantly stirred to encourage aggregation (43-46). v) In the magnetic technique, cells with a 2D structure are incubated inside a specific apparatus equipped with magnetic nanoparticles. These have the function of binding in a non-specific electrostatic manner to the cell membrane, thus affecting its magnetization. Once magnetized, the cells are enzymatically dissociated, inserted into a 3D apparatus, and subjected to a further magnetic field generated through different techniques (such as levitation, ring formation, or bioprinting), thus inducing aggregation and generation of 3D structures (47). vi) In the microfluidic technique, a device consists of two states of polydimethylsiloxane (PDMS), of which the microfluidic channel constitutes the upper one, while the lower one presents the cell culture chambers. The size of the spheroid will thus depend on the geometry chosen for the culture chambers (48). vii) The pellet culture method allows for the control of the size of the spheroid as a function of the number of cells that are placed in suspension (49).

**Figure 2:**
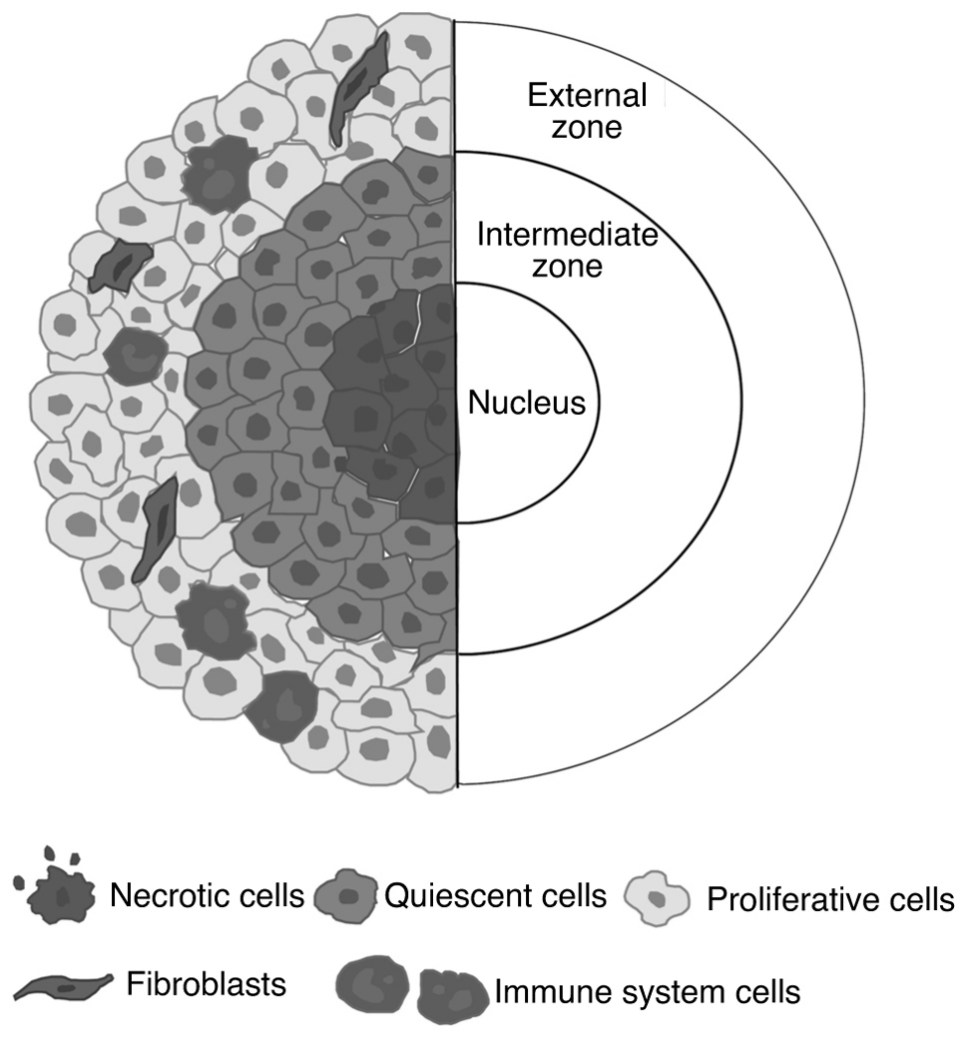
Structural organization of a spheroid, highlighting the main three layers: The nucleus, the intermediate zone and the external zone. On the left side, the distribution of various cell types, including necrotic, quiescent, proliferative, fibroblast and immune system cells is shown.

*Spheroids*. Spheroids are 3D cultures made of tumor cells that can assemble spontaneously into spheres during cell proliferation (50). Being cultured in ECM and the presence/absence of fibroblasts and immune cells allow the study of cell-cell and cell-ECM interactions (50). These structures generally have a diameter of 200 µm and have a spherical shape (50). Spheroids typically have a concentric cellular organization of three different layers: A nucleus comprised of necrotic cells, an intermediate cellular zone of quiescent cells, and finally, an external zone of proliferating migrating cells (50) (Fig. 2).

Spheroids represent one of the most commonly used 3D models in the study of tumor organization and functionality, being employed in the characterization of various tumor species such as breast, colon and different types of solid tumors. Depending on the cell type and preparation technique, tumor spheroids can be classified into four main classes, as follows: Tumorspheres obtained from solid tumors; tumorspheres derived from tissues remodeled following mechanical or enzymatic dissociation; multicellular tumor spheroids based on primary cell suspensions or cell lines; and finally, organotypic multicellular spheroids obtained from tumor fragment cultures (50-53).

One of the fundamental requirements during the design and creation of these models is to generate biomimetic prototypes that can model the interactions within the TME, as well as the control of the pharmacological action. Therefore, it is possible to state that these 3D tumor models can mimic cellular interactions through adhesion molecules and soluble factors, reproducing ECM with characteristics typically observed in the tumor condition (51).

Due to the difficulties encountered in faithfully reproducing the polymorphic characteristics of a tumor, increasingly complex spheroid models have been introduced. Beginning from simple monoculture, these models have allowed for the integration of heterotypic cells, ultimately leading to *ex vivo* models derived directly from patients. Although the majority of studies in the literature have focused on spheroids derived from monoculture, due to tumor complexity, it is always preferable to use a heterotypic platform capable of integrating different cell types (52).

*Organoids*. Organoids are multicellular structures generated from 3D cultures of stem cells *in vitro*. These cells can be embryonic, pluripotent, or adult tissue cells. Presenting the ability to self-assemble, they can exactly mimic the functionality of the originating tissue. Owing to these exceptional characteristics, the organoid model successfully maintains genetic and phenotypic stability (53). They can overcome both 2D and *in vivo* model limitations (54).

*In vivo* models, including patient-derived tumor cells (PDCs) and patient-derived xenografts (PDX), are often used as tumor models (55). PDCs are primary cell cultures obtained directly from tumor cells extracted from fluids or tissues; although they can replace the traditional tumor cell lines used during studies, their main limitation is that they are 2D models, therefore failing to faithfully reproduce the tumor architecture (56). The PDX model is generated using immunodeficient mice that are administered a graft of tumor cells extracted directly from the patient (57).

Although these models provide rather reliable similarities in the human component, they are associated with a series of issues, such as a low success rate, limited screening possibilities and relatively long implementation times. However, owing to their versatility, organoids are commonly used in a number of fields, such as oncology and microbiology.

One of the most promising applications of organoids and cancer organoids, the so-called tumoroids, derived from patients is certainly that of creating a biobank that allows for the implementation of personalized therapy based on pharmacological screening implemented on these models (58). To date, multiple available models of tumor organoids are used in tumor modeling, pharmacological screening, immunotherapy and precision medicine. These models have been applied in tumor forms such as bladder cancer, ovarian cancer, prostate cancer, liver cancer, breast cancer, colorectal cancer, lung cancer and pancreatic cancer (55).

Despite their interesting structure, organoids lack vascularization. Therefore, to correctly mimic highly vascularized tissues with a dynamic microenvironment, such as the brain and heart, it is essential to implement this feature. For this purpose, it is possible to use different vascularization techniques based mainly on the *in vivo* approach in an immunodeficient host (59,60).

Moreover, in the case of models aimed at studying pathologies such as infections, autoimmune components and cancer, it becomes essential to enrich the organoid with immune cells through epithelial organoid-immune cell coculture systems (61). Furthermore, by inserting innate immune cells into this model, it is possible to improve the degree of tumor attachment as well as the vascular inflammation typical of metastases (62).

### Scaffold-based 3D models: Organ-on-a-chip (OoC) and organ-on-a-plate

3D models equipped with scaffolds aim to reconstruct the dynamic interactions that are established between cells of the TME and the ECM (63). Compared with spheroids, 3D models equipped with scaffolds imitate the ECM and influence the mechanical and biochemical signals typical of cell-cell interaction. Therefore, these models represent excellent structures for cultivating primary tumor cultures, carrying out pharmacological safety and efficacy screening, and studying the role of stromal cells in cancer (64).

The biomaterials used in these models can be divided into the following main groups: i) Polymeric scaffolds are usually made in polymers, such as polyacrylamide, polylactin, poly (lactide-co-glycolic acid) or polyurethane (65). ii) Decellularized matrices are derived directly from diseased tissues or organs after the removal of cellular components, while preserving the ECM composition. This maintains the native structure, as well as the biomechanical characteristics, resulting in highly accurate models (66,67). iii) Hydrogels can be composed of natural gels, such as collagen and fibrinogen, synthetic polymers, or hybrids. Owing to their ability to mimic the ECM, hydrogels can allow the passage of soluble molecules, such as growth factors and cytokines, similar to tissues (68). iv) Hybrid scaffolds combine different materials, such as synthetic and natural polymers or hydrogels (69).

The devices used for these 3D cultures can include different models based on their fabrication. For example, they can be fabricated by solid freeform fabrication. This technique allows for the development of different types of scaffolds depending on the materials used, geometry and size of the pores. The 3D project is developed using software. Through computer-aided design technology, it is possible to better control manufacturing (70). This technique has aroused immense interest, particularly for its ability to create an architecture that imitates the native tissue microenvironment, as well as the interaction with the cells and components of the ECM (71).

Some of these are microfluidic devices comprise of circuits that allow continuous cell feeding via laminar flow. Through microfabrication, microchannels and micropillars <100 µm are generated, with an overall structure in the order of mm. This architecture, being equipped with a solid surface, mimics a physical environment such as the ECM. Moreover, the organization of channels, unlike other 3D culture technologies, allows for microfluidic devices to cultivate different cell types. The substrate used can be made of glass and/or silicon, polymeric material, or paper-based. Among the mainly used materials, PDMS is widely applied in biomedical technology. Due to its high biocompatibility and the possibility of modifying surface characteristics, such as chemical, physical and biological properties, exceptional applications can be obtained (72,73). Alternatively, devices for 3D cultures can be assembled manually to better exploit the properties of the chosen materials are exploited, in terms of biocompatibility to mimic intercellular interactions and the interaction with the matrix (71,72).

*OoC*. The first OoC was proposed in 2010 by Huh *et al* (74) as an artificial physiological system created on a chip. Depending on their functionalities and modalities, OoCs can be divided into single-OoCs, multi-OoCs (MOoCs), human-on-a-chip, and tumor-on-a-chip (ToC) (75).

The design of an OoC begins with a reductionist analysis of the target organ. It is essential to understand the structure of the target, simplifying its physiological functions. Subsequently, it is necessary to consider the selection of the cell culture, to control the dynamic flow perfusion, design the microstructure and account for the mechanical movement of biological organs (76).

**Figure 3:**
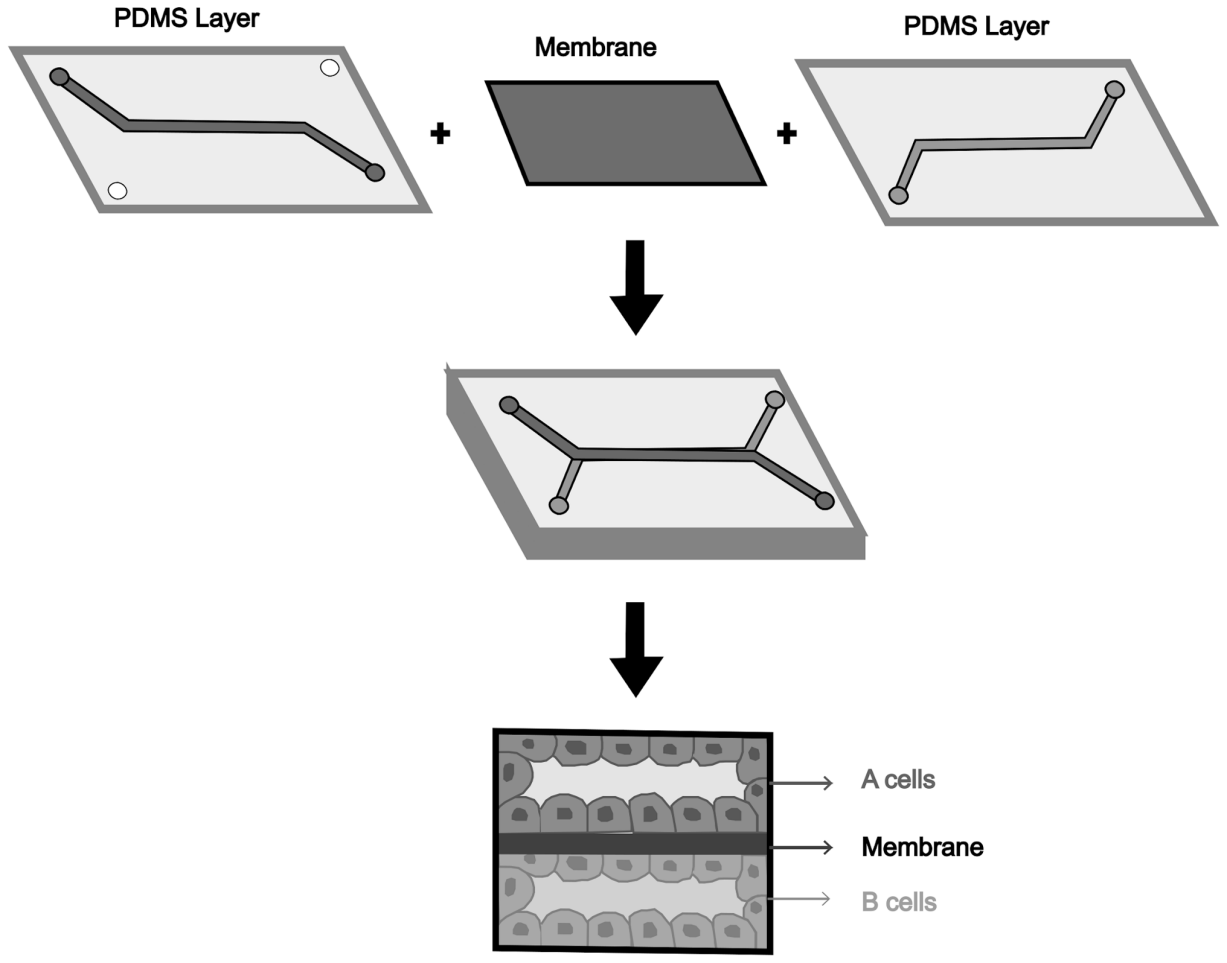
Example of a two-channel organ-on-a-chip model detailing the microfluidic channels and the cellular organization on the membrane that divides the two chambers. PDMS, polydimethylsiloxane.

OoCs are comprised of a physical compartment in which the cells are confined in different microfluidic channels (Fig. 3). The latter is used for the administration and transport of substances and the evaluation of measurable signals. Owing to their integrated compartment, it is possible to evaluate cellular behavior and the response of the microenvironment of the organ to the stimuli provided. Within the channels of an OoC, it is possible to incorporate polymeric membranes, thus modeling tissue interfaces and imitating barrier functions. Furthermore, in certain cases, it is possible to establish a vascular structure, increasing the complexity of the system and, therefore, the representativeness of the model (77).

Different OoC designs are dependent on the type of organ to be studied and the required characteristics. Typically, the structural classification is made based on the number of channels present and their organization. Therefore, there are single-channel chips, double-channel chips, respectively parallel or sandwich type and multi-channel chips. The dimensions are in the order of 1 cm, and the two channels are connected by a porous membrane so that interphase interactions between cells of the same tissue can be studied. To control the entry and exit of the fluid used and the introduction of biological material, the structure is equipped with entrances and exits. As regards the materials used, glass represents one of the oldest products, while PDMS is the most commonly used material; furthermore, the use of thermoplastics is currently spreading (78).

OoC systems can replicate a single unit or can be interconnected with each other. Thus, they can be classified into single- or MOoCs. These microfluidic cultures can also enable the connection of multiple units, leading to the concept of a body-on-a-chip or the replication of a TME, resulting in a ToC:

*i) Single-OoCs*. Contrary to what might be assumed, ‘single’ does not mean these models are simple or have only one channel/chamber, but rather that they can replicate a single unit. Single-OoCs can indeed host very complex co-cultures and differentiated tissues and have been developed to study the microphysiology and pathophysiology of numerous diseases, such as viral infections (79-81), lung diseases (82), or intestinal disorders (83), as well as for toxicological studies (84). They for allow the maturation and function of microtissues and the emulation of the physiological microenvironment. Examples of OoC can include lung-on-a-chip, which can reproduce the lung microenvironment, as well as the air-liquid interface and the barrier function performed for inhalation agents and can be used to carry out comparison studies between smoking and non-smoking conditions (85); brain-on-a-chip, which could be used to better mimic the chemical and electrical conditions of the human nervous system; liver-on-a-chip to mimic the structure of liver lobules; kidney-on-a-chip for glomerular study. Other examples are skin-on-a-chip, uterus-on-a-chip and vessels-on-a-chip models (86,87).

*ii) Multiorgan- and body-on-a-chip*. Developments in the field of OoCs have also led to the establishment of MOoCs. The connection of individual 3D organ models has allowed for the study of pharmacokinetics and pharmacodynamics, thus monitoring the intricate interactions between organs and studying their responses to drugs. MOoCs can also emulate blood circulation, allowing for the study of the dynamic response of the organs, in addition to the chronic cellular responses and the interaction between different organs in complex processes, such as absorption, distribution, metabolism and excretion (88). Through OoC, it is possible to generate different tissues capable of mimicking the complex functioning of the human body. These multi-tissue systems can be created with entirely human cells, becoming important metabolization models (89).

*iii) ToC*. The concept of a ToC involves creating a microfluidic 3D system that accurately mimics the behavior, biological activities, mechanical properties and various responses of tumor cells (90-92). One of the great advantages of the ToC is having both normal and cancer cells in the same model, thus better replicating the complex TME and allowing more accurate predictions of drug efficacy and toxicity, leading to better-informed decisions in both preclinical and clinical settings. This technology has been applied to a wide variety of tumors, such as pancreatic tumors (93,94), lung (95-97), brain (98,99) and blood (98,100-102). These advanced devices are particularly valuable in the context of breast cancer research; Chen *et al* (103) employed this technology for drug screening by evaluating the effects of different chemotherapeutic agents on the tumor model.

By understanding the factors that induce resistance in tumor cells, researchers can discover novel therapeutic strategies to combat disease. This knowledge may help to reduce the risks associated with current treatments. In addition, when primary cells from patients are used, this may pave the way for the development of personalized drugs, which represent the next frontier in cancer treatment (104).

*Organ-on-a-plate*. As with OoC systems, also known as organ plate models, organ-on-a-plate is an advanced approach within the field of tissue engineering and microfluidics that aims to replicate human organ functions in a laboratory setting. Previous studies have utilized this platform to perform high-throughput analysis for various physiological processes, such as: i) T-cell extravasation under flow: This process was analyzed using organ-on-a-plate technology, demonstrating its capability to model immune cell dynamics within the vascular system (105); ii) endothelial cell permeability: Researchers assessed the permeability of endothelial cells, which are crucial for understanding vascular barrier functions and pathological conditions, such as inflammation and cancer metastasis (106); iii) intestinal tract epithelium integrity: This technology has been employed to study the integrity of the intestinal epithelium, providing insight into gastrointestinal diseases and drug absorption (107).

This technology was also used for studying metastatic models, mimicking the early stages of breast cancer metastasis, including tumor cell invasion and intravasation (108). Utilizing this platform, a library of 86 targeted anticancer drugs with annotated molecular targets was screened. The results demonstrated the suitability of the platform for preclinical drug testing and drug discovery (108). Moreover, the metastasis-on-chip platform can be integrated with other established assays, such as tumor growth or motility assays, to enhance drug profiling outcomes (108). The development of automated imaging and analysis methods for the metastasis-on-chip platform has enabled high-throughput phenotypic drug screening. This automation not only improves the efficiency and reproducibility of experiments, but also allows for the detailed and comprehensive analysis of drug effects on cancer cell behavior.

All the aforementioned models, the pros and cons of which are highlighted in Table II, have contributed to research on drug resistance mechanisms, specifically in resistance mechanisms toward TKIs. TKIs represent a class of targeted cancer therapies that have revolutionized treatment since their discovery. Therefore, the present review discusses which models have provided insight into TKI resistance. It should be noted that while some studies highlight the general benefits of each 3D model type, scaffold-free and scaffold-based, they often do not address TKI-specific dynamics. This could indeed be an intriguing area for future research, possibly warranting a dedicated study to explore these unique applications in depth.

## 4. Cases of tyrosine kinase inhibitor resistance addressed using 3D models

TKIs specifically target enzymes known as tyrosine kinases, which play a crucial role in regulating cell growth, proliferation and survival pathways. The development of imatinib in the late 1990s marked a significant milestone in TKI discovery. Initially approved for the treatment of chronic myeloid leukemia (CML) (109), imatinib paved the way for subsequent TKIs targeting various tyrosine kinases implicated in various types of cancer (110). TKIs usually inhibit the enzymatic activity of tyrosine kinases by binding to their ATP-binding sites, thereby disrupting downstream signaling pathways involved in cancer progression. They are utilized in the treatment of various malignancies, including CML, gastrointestinal stromal tumors (GISTs) (111), non-small cell lung cancer (NSCLC) (112) and renal cell carcinoma (RCC) (113), among others. TKIs have demonstrated notable efficacy, significantly improving the outcomes and quality of life of patients.

Despite their therapeutic benefits, TKIs are not without limitations. One major challenge is the development of resistance (114,115), either through acquired mutations in the target kinase or the activation of alternative signaling pathways, leading to disease progression and treatment failure. Additionally, TKIs may exhibit off-target and adverse effects (114,116), impacting patient tolerability and treatment adherence. Addressing these limitations remains a key focus in oncology research, with ongoing efforts aimed at developing next-generation TKIs with improved efficacy, selectivity and safety profiles, as well as exploring combination therapies and personalized treatment approaches to overcome resistance and enhance clinical outcomes (117-120).

From a historical perspective, the development and introduction of imatinib and sorafenib were pivotal in the utilization of TKIs. These drugs marked significant advancements in targeted cancer therapies, laying the foundation for subsequent innovations in the field.

Imatinib, targeting BCR-ABL, KIT and PDGFR, has been FDA-approved for the treatment of CML and GISTs (121-124). Research has revealed that imatinib improves endothelial barrier stability and decreases invasive tumor cell activity (108). Ozer *et al* (108) explored their 3D model platform, metastasis-on-chip, for investigating cancer metastasis biology, drug discovery and drug reactions, providing potential for tailored anti-metastatic treatments for patients with triple-negative breast cancer. Imatinib notably decreased the intravasation of tumor cells, suggesting that its anti-metastatic effects may be linked to tumor-endothelial cell interactions. It enhanced the integrity and permeability of HUVEC monolayers when co-cultured with tumor cells/fibroblasts, mitigating endothelial barrier disruption and impeding tumor cell intravasation and extravasation (108).

Sorafenib is a multikinase inhibitor that targets several kinases, including Raf kinase (125-129), vascular endothelial growth factor receptor (VEGFR) (126,127) and platelet-derived growth factor receptor (PDGFR) (50,130,131). It is primarily utilized in the treatment of liver (129,132-135), kidney (126,136-140) and thyroid cancers (130,141-146). In this context, Ek *et al* (147) investigated the effects of sorafenib on a 3D cancer model of hepatocellular and colorectal carcinoma, identifying oxidative phosphorylation (OXPHOS) as a target vulnerability. They assessed global transcriptional responses in monolayer cell cultures, multicellular tumor spheroids, and tumoroids generated from a colorectal carcinoma patient. Cells in 3D models were sensitive to OXPHOS inhibitors, but resistant to other TKIs and chemotherapeutic drugs. Sorafenib and nitazoxanide exhibited an additive effect in reducing viability, regrowth potential, and inhibiting mitochondrial membrane potential at clinically relevant concentrations (147). An additional aspect of sorafenib resistance was investigated by Bielecka *et al* (148) in RCC cells treated with axitinib and sorafenib. Since hypoxia is known to reduce the efficacy of chemotherapy in solid tumors, Bielecka *et al* (148) developed a 3D model with suspended culture to mimic the molecular pathways characteristic of hypoxic conditions and the cell-cell dynamics found in the tumor environment. This model confirmed that resistance is linked to low oxygen levels, which induce conformational changes in the expression of key proteins, fulfilling TKI resistance (148).

Likewise, over the past years, sunitinib has been widely employed in RCC (kidney cancer) (149-152), GISTs (153-158) and pancreatic neuroendocrine tumors (159-162). Polena *et al* (163) investigated the effects of sunitinib, combined with bevacizumab, on VE-cadherin, a protein crucial for endothelial integrity. Using an endothelial monolayer model and both homotypic and heterotypic 3D cell models that mimic tumor growth, they assessed the effects of sunitinib and the antibody bevacizumab on VE-cadherin modifications and patient outcomes (163). The results revealed that sunitinib directly targeted VE-cadherin, inhibiting its phosphorylation and cleavage, and reducing endothelial cell migration in the 3D models. In patients with metastatic RCC, the baseline levels of soluble VE-cadherin (sVE) were higher than those in healthy donors. A decrease in sVE levels after 4 weeks of treatment was observed in responders to sunitinib, but not to bevacizumab (163). These findings obtained owing to the faithful 3D model employed suggest that sVE levels may be a valuable biomarker for monitoring the efficacy of targeted therapies, such as sunitinib in patients with cancer (163).

Sunitinib was also investigated in the study by Rausch *et al* (164), who conducted a study on clear cell RCC, revealing that resistance to sunitinib involves complex molecular changes, including dysregulated cell cycle progression and lysosomal sequestration of the drug. Using 3D heterotypic co-cultures, the researchers could mimic resistance mechanisms and test an optimized multidrug combination. This approach reduced metabolic activity in resistant cancer cells by >80%, highlighting the importance of 3D models in studying and overcoming TKI resistance, providing a more accurate platform for evaluating drug responses and developing effective treatments (164).

As regards more recent TKIs, the gefitinib inhibitor of the epidermal growth factor receptor (EGFR) tyrosine kinase was intensely studied in a 3D model. It is primarily used in the treatment of NSCLC with specific EGFR mutations. In the 3D lung carcinoma model, Stratmann *et al* (165) were able to prove the efficacy of TKIs, particularly gefitinib, in targeting EGFR. Employing 3D models on decellularized tissue matrices, the researchers created a complex TME that more accurately reflected clinical conditions. This approach revealed notable differences in drug responses when compared to conventional 2D cultures. In 3D models, gefitinib effectively inhibited EGFR activation in HCC827 cells, which harbor an activating EGFR mutation, leading to decreased proliferation and increased apoptosis (165). The 3D models enabled the quantitative measurements of proliferation, apoptosis and invasion, exhibiting strong associations with both *in silico* EGFR signaling models and clinical data. This thorough analysis established a reliable platform for assessing drug efficacy and tumor behavior. Additionally, applying TGFβ1 in the 3D model triggered EMT and tumor cell invasion, as evidenced by changes in cell morphology and EMT marker expression, effects not observed in 2D cultures, highlighting the enhanced predictive accuracy of 3D models (165).

Moreover, the efficacy and resistance of a number of gefitinib combinations were previously explored in 3D models. Liu *et al* (166) investigated the resistance mechanisms of lung cancer cells to EGFR-TKIs. The loss of wild-type EGFR reduced cell proliferation, migration and 3D-spheroid formation, while the loss of mutant EGFR or TKI resistance enhanced these processes. Wild-type EGFR disruption suppressed HER2/HER3 expression, but mutant EGFR loss or TKI resistance increased HER2/HER3 expression, promoting tumor cell survival and drug resistance via cyclin D1. Inhibiting cyclin D1/CDK4/6 re-sensitized erlotinib-resistant cells to erlotinib. Cyclin D1 expression in patients with NSCLC was associated with a worse survival (166). A 3D-spheroid tumor model was used to mimic the tumor microenvironment. In EGFR wild-type cell lines (A549 and H1299), EGFR ablation significantly inhibited cell proliferation, migration and tumor formation. Conversely, EGFR19del and erlotinib-resistant cells (H1650 and HCC827) exhibited increased proliferation, migration and 3D-spheroid formation upon EGFR19del ablation or TKI resistance (166). Another example of investigating the TKIs, gefitinib and erlotinib, is represented in the study conducted by Jacobi *et al* (167) that used 3D spheroids and organoids created with the hanging drop method embedded in Matrigel/collagen type I matrices. That study demonstrated the significant advantages of using 3D lung cancer cell cultures, specifically spheroid models, over traditional 2D cultures in proving the efficacy of TKIs targeting EGFR signaling. The 3D spheroid models revealed drug sensitivities that more closely match clinical outcomes. Notably, the addiction to the EGFR oncoprotein, crucial for the effectiveness of EGFR inhibitors, was observed only in 3D spheroid cultures. Furthermore, HCC827 cells with EGFR mutations exhibited significant sensitivity to EGFR inhibitors in 3D spheroids, but not in 2D cultures (167). Using the same cancer model, Zheng *et al* (168) investigated the novel STAT3 inhibitor, W2014-S, for its potential to enhance the efficacy of gefitinib and erlotinib in overcoming drug resistance in NSCLC. The study by Zheng *et al* (168), which included *in vivo* experiments using human NSCLC cell xenografts and PDX in mouse models, demonstrated that W2014-S enhanced the antitumor effects of gefitinib. By disrupting STAT3 signaling, a pathway often activated in NSCLC and associated with drug resistance, W2014-S effectively inhibited cancer cell proliferation, survival, migration, and invasion in 3D cultures. Additionally, it significantly sensitized TKI-resistant NSCLC cells to gefitinib and erlotinib, highlighting the importance of 3D cell cultures in evaluating the efficacy of new treatments (168).

In another cancer model, gefitinib and erlotinib were investigated by Hoque *et al* (169), who demonstrated that these TKIs more effectively inhibited cell viability, clonogenic growth and wound healing in human squamous epithelial A431-A6 cells compared to the controls in spheroids. Moreover, the study showed that increased AnxA6 levels enhance TKI efficacy by suppressing A431 cell motility and invasiveness. Likewise, in renal cancer cells, Fu *et al* (170) used 3D cultures to demonstrate the efficacy of TKIs, specifically gefitinib and erlotinib, targeting EGFR signaling. By employing spheroids and organoids, they found that these models reveal drug sensitivities that more closely align with clinical outcomes compared to traditional 2D cultures. The addiction to the EGFR oncoprotein, crucial for the effectiveness of EGFR inhibitors, was evident only in 3D cultures (170). Furthermore, 3D models were more predictive of clinical responses, as observed with HCC827 cells exhibiting significant sensitivity to EGFR inhibitors in 3D, but not in 2D cultures (170). The study Fu *et al* (170) underscores the enhanced predictability and clinical relevance of 3D models, advocating for their use in drug development and personalized cancer treatments to improve therapeutic outcomes (170).

As regards other combinations of gefitinib, Liu *et al* (171) explored the combination of gefitinib with rapamycin to address gefitinib resistance in NSCLC cells. The results of their study revealed that this combination significantly improved therapeutic outcomes, as rapamycin activated autophagy, synergizing with gefitinib to reduce cell viability and tumor formation both *in vitro* and *in vivo*. Additionally, an anti-EGFR aptamer-functionalized nanoparticle system was developed for targeted delivery, enhancing the cytotoxic effects of the drug combination (171).

Further combinations of other EGFR-TKIs were studied in 3D cell cultures of NSCLC. Kim *et al* (172) investigated the role of the receptor tyrosine kinase, AXL, in mediating resistance to EGFR-TKIs, including the third-generation TKI, osimertinib. Using both *in vitro* and *in vivo* models, including 3D cell cultures, xenograft tumors and PDX, their study found that AXL overexpression contributes to resistance by extending the protein degradation rate. Targeting AXL degradation with yuanhuadine (YD), an AXL degrader, restored sensitivity to EGFR-TKIs. The combination of YD and EGFR-TKIs effectively delayed or overcame resistance in EGFR-mutant NSCLC cells (172).

A more comprehensive approach to EFGR inhibition was offered by the study conducted by Hou *et al* (173), which used three-dimensional convolutional neural networks (CNN) with deep transfer learning to predict the treatment response in patients with stage IV lung adenocarcinoma with EGFR mutations. The 3D CNN model was able to stratify patients into subgroups with different progression risks and predict progression-free survival more accurately than models based solely on clinical features. This demonstrates the importance of 3D models in proving the efficacy of TKIs, providing more precise and reliable tools for clinical decision-making (173).

As regards fibroblast growth factor receptor (FGFR) inhibition, a number of TKIs inhibiting FGFR1 have emerged as a promising strategy for cancer therapy. A novel *in vitro* 3D culture system was developed by Ko *et al* (174) to study FGFR signaling in prostate cancer stem cells within cell lines (PC3, DU145 and LNaP), and the induced pluripotent iPS87 cell line. This 3D model, which exhibited increased stemness markers, demonstrated that the desired inhibition, with BGJ398 or dovitinib, effectively reduced cell survival and proliferation. The use of the spheroids was crucial in demonstrating the efficacy of FGFR1-targeting TKIs, providing a more realistic representation of tumor behavior and drug response compared to 2D monolayer cells (174).

Finally, although resistance against TKIs is usually studied in cancer cell lines, they are not able to widely recapitulate patient-cancer model complexities. In the realm of lung cancer research, Kim *et al* (175) emphasized the critical importance of patient-derived organoids (PDOs) in demonstrating the effectiveness of TKIs for advanced-stage lung adenocarcinoma. PDOs were established from patients with advanced-stage lung adenocarcinoma and analyzed for genetic alterations and responses to targeted therapies. These organoids retained somatic alterations, including driver mutations, from the original patient tumors, and their responses to TKIs mirrored the clinical outcomes of the corresponding patients. PDOs with EGFR exon 19 deletions and BRAF G464A mutations responded to dabrafenib/trametinib, and those with EGFR L747P mutations were sensitive to afatinib, consistent with patient responses (175). They were used to identify effective therapies for novel molecular targets, demonstrating the efficacy of poziotinib against ERBB2 exon 20 insertions and pralsetinib against *RET* fusions. The use of 3D models such has PDOs directly from patients thus provides a more accurate and clinically relevant platform for evaluating the efficacy of TKIs (175).

Currently, despite all the advantages previously listed, it is important to also acknowledge the limitations of 3D models that hinder clinical translation. A number of these models require specialized equipment and are costly, limiting accessibility. They also exhibit high variability and low standardization, which affect reproducibility and complicate large-scale studies. Furthermore, advanced analytical techniques are often required, rendering 3D models less suited for high-throughput screening.

Nevertheless, the crucial role of clinical validation in establishing the relevance of findings from 3D models is commonly acknowledged, particularly for studying resistance to TKIs. While the present review synthesizes current preclinical data, additional clinical research is essential to evaluate how insight from 3D models can translate into real-world settings. Further studies using patient-derived samples or clinical trials could bridge this gap and underscore the translational potential of 3D models in oncology. Although robust clinical validations are limited, early advancements demonstrate the potential of 3D models to refine TKI efficacy predictions and support personalized cancer therapies. Few established clinical validations directly address TKI resistance findings from 3D models; however, recent studies have shown promise.

For example, recent research has combined computational and mechanistic modeling with *in vitro* 3D cultures to predict patient-specific responses. This approach has shown potential in lung adenocarcinoma treatments with EGFR-targeting TKIs (176), such as osimertinib, where data from clinical trials (e.g., NEJ002-NCT00322452 and FLAURA-NCT02296125) has been used to align model predictions with actual patient outcomes (177).

These advancements provide a foundation for future research to further validate and strengthen 3D model findings within clinical settings. As development progresses, 3D models can also assist in refining dosage strategies and minimizing off-target effects by simulating drug behavior in a controlled microenvironment, thus bridging the gap between preclinical insights and clinical outcomes.

## 5. Application of 3D models in other cancer drug classes

Having explored the valuable role of 3D models in evaluating the efficacy of TKIs and understanding their limitations in complex environments, the present review now provides a brief overview of how 3D models have brought innovation to other cancer drug classes. These include chemotherapeutic agents, hormone therapies and immunotherapies.

Hirschhaeuser *et al* (178), in a comprehensive review, discussed the benefits of using 3D spheroid models to simulate *in vivo*-like conditions for chemotherapy studies. These models allow for a better understanding of drug penetration, efficacy and resistance, as they replicate the architecture and gradients present in tumors. Wenzel *et al* (179) introduced a 3D platform to test a variety of compounds targeting dormant tumor cells, validating a screening system for substances that would not be adequately evaluated using traditional 2D models. Additionally, Imamura *et al* (180) evaluated the response of breast cancer cell lines cultured in 3D to assess drug sensitivity to paclitaxel, oxygen status, and significant markers of proliferation (181), such as Ki-67 and cell death, such as caspase. They concluded that the 3D model more accurately simulated *in vivo* tumor characteristics, revealing drug resistance mechanisms linked to hypoxia, dormancy and anti-apoptotic features (180).

Hormone therapies, such as tamoxifen, are widely used in the treatment of hormone receptor-positive breast cancer. Leeper *et al* (182) determined the sensitivity of breast cancer tissues to tamoxifen using a collagen-based 3D culture. The model shown by Leeper also demonstrated the histopathological changes caused by the drug, offering a more precise evaluation than traditional methods (182). Similarly, enzalutamide, a key drug in prostate cancer therapy, modulates androgen receptor activity as shown by Andolfi *et al* (183). The study demonstrate that using a 3D model allowed for clear evidence of reduced cell proliferation when MED12 and CDK8/19 were inhibited by enzalutamide, offering valuable insights into prostate cancer treatment (183).

Jenkins *et al* (184) highlighted how 3D models have been particularly useful in studying immune checkpoint inhibitors such as PD-1/PD-L1 and CTLA-4. These models enable researchers to recreate immune cell-tumor interactions in a more realistic tumor microenvironment, helping to elucidate mechanisms of immune resistance and response (184).

3D models have revolutionized cancer research by providing more physiologically relevant systems for evaluating drug efficacy, toxicity, and resistance mechanisms. While the present review has focused on the use of 3D models in studying TKIs, their application extends to other major drug classes, including chemotherapeutics, hormone therapies, immunotherapies and anti-angiogenic agents. These models enable researchers to capture complex tumor microenvironment dynamics that are often missed in 2D cultures, ultimately improving the translation of preclinical findings to clinical outcomes. Expanding the use of 3D models across different drug classes can lead to more effective cancer therapies and a better understanding of treatment responses.

## 6. Conclusion and future perspectives

The adoption of 3D models has revolutionized the strategies used to test chemotherapeutic agents, providing a more realistic representation of the TME and its complexities. These models have enhanced predictive power and accuracy in assessing drug efficacy and toxicity, streamlining the drug development process and reducing reliance on traditional 2D cultures. Researchers are now equipped with more robust platforms to inform clinical decision-making and treatment strategies.

Looking ahead, the continued refinement of 3D models holds immense potential for overcoming the limitations associated with TKIs. These models provide valuable tools for investigating interactions between cancer cells, stromal components, and immune cells within the TME. By unraveling the complex mechanisms behind TKI resistance, novel therapeutic strategies can be developed to enhance drug efficacy and overcome resistance barriers.

One exciting future direction is the integration of 3D models with multi-omics approaches, including genomics, transcriptomics, proteomics and metabolomics (185). By coupling these models with artificial intelligence (AI) and machine learning algorithms, researchers could gain deeper insight into tumor biology and drug responses (186). This integration would allow for the real-time monitoring of molecular changes in response to treatments, providing predictive data that could help preempt resistance and suggest alternative therapeutic pathways. These advancements have the potential to reshape the drug development pipeline by significantly improving the early detection of drug efficacy or failure, reducing the time and cost associated with clinical trials.

As 3D models continue to evolve, their integration with cutting-edge technologies, such as multi-omics, AI, immune-oncology models and microfluidics will push the boundaries of cancer research and therapy. These advancements will contribute to more personalized and precise treatment regimens, reshaping the landscape of oncology. Collaborative efforts across disciplines, driven by innovative technological developments, will not only enhance the understanding of cancer biology, but may also improve the translation of preclinical findings into clinical success. Ultimately, this progress promises to deliver more effective cancer therapies, ushering in a new era of precision oncology for patients worldwide.

## Figures and Tables

**Table I:** Limitations and implications of 2D models.

Limitation of 2D models	Implications	(Refs.)
Evaluation of pharmacokinetics	Often neglect crucial aspects such as drug metabolism, distribution, and excretion	(187)
Failure to replicate drug resistance mechanisms	Resistance to chemotherapy via various pathways, including genetic mutations, epigenetic alterations and interactions with the surrounding microenvironment may not be faithfully represented	(3)
Lack of physiological gradients	Poor understanding of drug effects on complex biological systems	(4-6)
Erroneous predictions of drug effectiveness	Diminished predictive precision concerning drug reactions and adverse effects in humans	(5,6)
Lack the three-dimensional arrangement and cellular interplay	Consequently, there is no prove of the interactions with extracellular matrix, immune cells and blood vessels affecting the study of drug interactions in a physiologically relevant context	(7-10)
Lack of dimensionality of the extracellular matrix surrounding cells	It profoundly influences cell proliferation, differentiation, cell survival and cellular responses to external stimuli and challenges	(11-15)
Tumorigenesis and metastasis are oversimplified in monocultures	The study of metastasis process cannot be reduced to cell movement through a wound healing assay. It should also include evaluating whether these cells can invade tissues or/and enter vessels	(27)
False positives or negatives may occur in drug screening	Simplistic 2D models fail to translate to success in more complex *in vivo* models or clinical trials	(29)
Poorly understood interactions between malignant cells and stromal components within the tumor microenvironment	These elements are crucial in influencing tumor initiation, progression, and response to treatment	(35)

**Table II:** Pros and cons of 3D models per cancer type.

3D models	Pros	Cons	Cancer types
Spheroids	Imitate cellular interactions with adhesion molecules and soluble factors; Reproduce the tumor ECM (51).	Difficulty in reproducing the polymorphic characteristics of the tumor (52).	Breast, colon and different types of solid tumors (50-53).
Organoids	Overcome 2D and *in vivo* models limitations (54); great versatility, as tumor modeling, drug screening, immunotherapy and precision medicine (55,58).	Lack of vascularization (59,60).	Bladder, ovarian, prostate, liver, breast, colorectal, lung cancer pancreatic cancer (55).
Organ-on-a-chip	Increasing the complexity of the system and the representativeness of the model (77); possibility of comparing tumor cells and healthy cells in the same model (90-92).	Issue in identifying the biological target; difficulties to simplify its physiological functions by designing the microstructure (76).	Pancreatic tumors (93,94), lung tumors (95-97), brain tumors (98,99), blood cancer (98,100-102), breast cancer (103).
Organ-on-a-plate	Possibility of studying multiple physiological processes and particularly metastatic models (105-108).	Need to increase the reproducibility of the experiment (108).	Breast cancer metastasis (108).

The numbers in parentheses are the reference citations.

## Data Availability

Not applicable.
